# Efficacy Comparison of Repeated Low-Level Red Light and Low-Dose Atropine for Myopia Control: A Randomized Controlled Trial

**DOI:** 10.1167/tvst.11.10.33

**Published:** 2022-10-21

**Authors:** Yanxian Chen, Ruilin Xiong, Xu Chen, Jian Zhang, Gabriella Bulloch, Xiaoxuan Lin, Xiaoman Wu, Jinying Li

**Affiliations:** 1 Department of Ophthalmology, Peking University Shenzhen Hospital, Shenzhen Peking University–The Hong Kong University of Science and Technology Medical Center, Shenzhen, China; 2 State Key Laboratory of Ophthalmology, Zhongshan Ophthalmic Center, Sun Yat-sen University, Guangdong Provincial Key Laboratory of Ophthalmology and Visual Science, Guangzhou, China; 3 Centre for Eye Research Australia, Melbourne, Australia; 4 Ophthalmology, Department of Surgery, University of Melbourne, Melbourne, Australia

**Keywords:** red light, atropine, myopia progression, axial length, randomized controlled trial

## Abstract

**Purpose:**

To compare the treatment efficacy between repeated low-level red light (RLRL) therapy and 0.01% atropine eye drops for myopia control.

**Methods:**

A single-masked, single-center, randomized controlled trial was conducted on children 7 to 15 years old with cycloplegic spherical equivalent refraction (SER) ≤ −1.00 diopter (D) and astigmatism ≤ 2.50 D. Participants were randomly assigned to the RLRL group or low-dose atropine (LDA, 0.01% atropine eye drops) group and were followed up at 1, 3, 6, and 12 months. RLRL treatment was provided by a desktop light therapy device that emits 650-nm red light. The primary outcome was the change in axial length (AL), and the secondary outcome was the change in SER.

**Results:**

Among 62 eligible children equally randomized to each group (31 in the RLRL group, 31 in the LDA group), 60 children were qualified for analysis. The mean 1-year change in AL was 0.08 mm (95% confidence interval [CI], 0.03–0.14) in the RLRL group and 0.33 mm (95% CI, 0.27–0.38) in the LDA group, with a mean difference (MD) of −0.24 mm (95% CI, −0.32 to −0.17; *P* < 0.001). The 1-year change in SER was −0.03 D (95% CI, −0.01 to −0.08) in the RLRL group and −0.60 D (95% CI, −0.7 to −0.48) in the LDA group (MD = 0.57 D; 95% CI, 0.40–0.73; *P* < 0.001). The progression of AL < 0.1 mm was 53.2% and 9.7% (*P* < 0.001) in the RLRL and LDA groups, respectively. For AL ≥ 0.36 mm, progression was 9.7% and 50.0% (*P* < 0.001) in the RLRL and LDA groups, respectively.

**Conclusions:**

In this study, RLRL was more effective for controlling AL and myopia progression over 12 months of use compared with 0.01% atropine eye drops.

**Translational Relevance:**

RLRL therapy significantly slows axial elongation and myopia progression compared with 0.01% atropine; thus, it is an effective alternative treatment for myopia control in children.

## Introduction

Myopia is characterized by excessive axial length and has become a leading cause of vision impairment worldwide.^1^^–^^3^ It is estimated that myopia will affect up to 50% of the global population by 2050,^4^ and its economic burden will likely reach $1.7 trillion.^5^ Myopia often progresses in early childhood and teenage years and if not controlled can cause vision-threatening complications, including retinal detachment, glaucoma, cataract, and macular degeneration.^6^^–^^8^ Myopia has a significant effect on children, as it affects each child personally and influences public health at a global scale.

Atropine eye drops are known to slow myopia progression by approximately 30% to 80%, but side effects such as photophobia, nearsighted blurry vision, and rebound effect are commonly seen in higher dosages.^9^^–^^11^ At lower doses, pupil size and accommodation are affected much less, and axial length is controlled more effectively than at higher doses, albeit side effects are still problematic.^11^ Despite this, atropine eye drops are a first-line therapy for controlling myopia progression because there are currently few alternatives with better safety profiles.^12^^–^^14^

Repeated low-level red light (RLRL) therapy has been used to treat amblyopia in children^15^ but was recently repurposed for myopia control. A multi-center randomized control trial (RCT) reported a 69.4% reduction in axial length progression and a 76.6% reduction in myopia progression in children using RLRL therapy compared with wearing spectacles only.^16^ A similar effect was observed after a 9-month follow-up in a retrospective study.^17^ When Xiong et al.^18^ compared RLRL with orthokeratology, a more profound effect on axial length (AL) control was evident after RLRL treatment for 6 months. In these studies, a 650-nm, single-wavelength red light was used, and the protective effect appears to be associated with an increase in choroidal thickness. With RLRL now being considered to be among the popular myopia therapies, a comparison with low-dose atropine is appropriate. Herein, we report the findings of a 12-month RCT comparing RLRL with low-dose atropine (LDA) with regard to their control of myopia in children.

## Methods

### Study Design

We conducted a 12-month, single-center, parallel-group randomized controlled trial to compare the efficacy of RLRL therapy versus 0.01% atropine for myopia control at Peking University Shenzhen Hospital. All examinations were performed according to the study protocol and used only approved equipment. The study was conducted in accordance with Good Clinical Practice guidelines, and adhered to the tenets of the Declaration of Helsinki. Written informed consent was obtained from all participants, their parents, or statutory guardians. Ethics approval was obtained from the ethics committees at Peking University Shenzhen Hospital. The trial has been registered with the Chinese Clinical Trial Registry (ChiCRT2100045834).

### Eligibility

The study included participants 7 to 15 years old with myopia greater than −1.0 diopter (D) and cycloplegic spherical equivalent refraction (SER) ≤ −1.00 D, astigmatism ≤ 2.50 D, and best-corrected visual acuity (BCVA) of 1.0 or 20/20 or better in both eyes and who agreed to accept random allocation to the treatment groups. The study excluded children who had undergone previous myopia control treatments, including atropine eye drops (0.01%–0.5%) or orthokeratology, or who had anisometropia > 1.50 D, congenital ocular abnormalities, myopia secondary from other conditions (e.g., retinopathy of prematurity), media opacity in the eye, history of refractive surgery or intraocular surgery, or a condition where active inflammation occurred on the ocular surface.

### Randomization and Masking

Computerized random number generation was performed by a masked statistician not affiliated with the study site, and treatment assignment was sealed in opaque envelopes. Each eligible participant was randomly assigned to either the RLRL group or the 0.01% atropine eye drops group according to what the envelope revealed. Due to the nature of the intervention, parents and children were not masked to treatment allocations, but the technicians and optometrists who assessed the treatment outcome were masked.

### Study Procedures

The intervention group received RLRL therapy with a table-mountable commercially available device (Eyerising; Suzhou Xuanjia Optoelectronics Technology, Jiangsu, China). The device emits red light at 650 ± 10 nm from semiconductor laser diodes at an illuminance level of 1600 lux from pupil to fundus. The device was given free of charge to the children, and children and parents were instructed to use the RLRL therapy for 3 minutes twice daily, with at least 4 hours between sessions, for 7 days a week until the last follow-up visit. The process of treatment is shown in Supplementary Figure S1, where the subject is seen sitting in front of the device with both eyes open during the RLRL therapy. The comparison group received 0.01% atropine eye drops (Shenyang Xingqi Pharmaceutical Co., Ltd., Liaoning, China) in both eyes at bedtime every day until the last follow-up visit. The eye drops were delivered to children in 0.4-mL disposable containers. Participants were instructed to wear single-vision spectacles throughout the study. All participants were instructed by one of the investigators (YC or JL) to use the RLRL therapy or atropine eye drops following the protocol provided.

All participants underwent baseline examinations and were followed up at 1, 3, 6, and 12 months. Cycloplegia was conducted with three drops of 1% cyclopentolate (Alcon, Geneva, Switzerland) at baseline and at 6 and 12 months. Refraction was measured using an autorefractor (ARK-1; Nidek, Inc., San Jose, CA) after full cycloplegia was achieved (diameter of pupil was ≥6 mm and pupil light reflex was absent). At least three consistent readings of cycloplegic refractive error were recorded. The difference in spherical or cylindrical power among the three readings was required to be within 0.25 D, and the average of these three readings was used in the analysis.

Ocular biometry was collected at each visit using the IOLMaster 500 (Carl Zeiss Microscopy, Jena, Germany) before cycloplegia. Average ALs with at least five measures within ≤0.05-mm difference were recorded. Corneal curvature (CC), anterior chamber depth (ACD), and white-to-white (WTW) corneal diameter were also measured. Uncorrected visual acuity (UCVA) and BCVA were assessed using the Early Treatment Diabetic Retinopathy Study E chart (Precision Vision, Woodstock, IL) at a distance of 4 m. The vision test protocol was the same as that found in the Refractive Error Study in Children.^19^ In brief, the test began at the top line (20/200) and continued to the line below if at least four of the five optotypes were correctly recognized. The lowest line read successfully was recorded as the visual acuity for the eye. Intraocular pressure (IOP) was measured three times per eye using non-contact tonometry (TX-20 Full Auto Tonometer; Canon, Tokyo, Japan), and the average was used for analysis.

### Outcomes

The primary outcome was change in AL from baseline to the 12-month follow-up. Key secondary outcomes were the 1-year changes in SER, CC, ACD, and WTW corneal diameter. Other prespecified secondary outcomes included 1-year changes in UCVA, BCVA, and IOP. Adverse events were assessed and registered at every visit, including but not limited to a sudden vision loss of two lines or more, a scotoma, photophobia, allergy, dry mouth, or tachycardia. The individual treatment would be stopped if a child experienced a severe adverse event, such as blindness, death, hospitalization, or conditions requiring medical or surgical interventions.

### Monitoring Treatment Compliance

We monitored the compliance of treatment of participants randomized to RLRL therapy or 0.01% atropine eye drops. In the RLRL group, the frequency of RLRL therapy was automatically recorded by the online management system connected to the device. The compliance of RLRL therapy was calculated as the percent of completed RLRL therapy sessions among the total therapy sessions a participant was anticipated to complete in 1 year. In the atropine treatment group, all participants were asked to bring back the used eye drop containers at each follow-up visit (Supplementary Fig. S2). For those who forgot to bring back the containers, we asked the parents to report the remaining eye drops at home. Compliance for the 0.01% atropine eye drops was calculated as the percent of empty containers among the total distributed containers.

### Statistical Analysis

Based on previously published data, the mean annual AL progression in children 7 to 15 years old who received the treatment of 0.01% atropine was expected to be 0.30 mm.^11^ We assumed that the progression of AL in the RLRL group was 0.10 mm per year, with a pooled standard deviation of 0.18 mm. Based on these assumptions, sample size for outcome analysis was estimated to be 24 subjects per group (48 subjects in total) to achieve 80% power at an α level of 0.05. After adjusting for a 15% loss to follow-up, the total sample size for enrollment was 56 subjects. SER was calculated as the sum of sphere and 1/2 cylinder. CC was calculated as the average of the greatest and least corneal curvatures measured by the IOLMaster. AL shortening was defined as a decrease of AL > 0.05 mm from baseline, exceeding the measurement error of the IOLMaster.^20^

Treatment efficacy for the comparison of primary and secondary outcomes between RLRL and 0.01% atropine was evaluated by longitudinal linear mixed-effects models. Data from both eyes of all children who completed one or more follow-up visits were included in the mixed-effects model. In each mixed-effects model, group, visit, and group-by-visit interactions were modeled as fixed effects, and baseline age, sex, and baseline measures of outcome were included as covariates. The subjects were included as a random factor within an unstructured covariance matrix, and eyes were considered as repeated measures within a compound symmetry structure matrix. Missing data at the specific follow-up visits were not imputed because linear mixed-effect models still provide valid results when data are missing at random. All of the analyses were conducted using Stata 16 (StataCorp, College Station, TX), and two-sided *P* < 0.05 was considered statistically significant.

## Results

**Figure 1.**
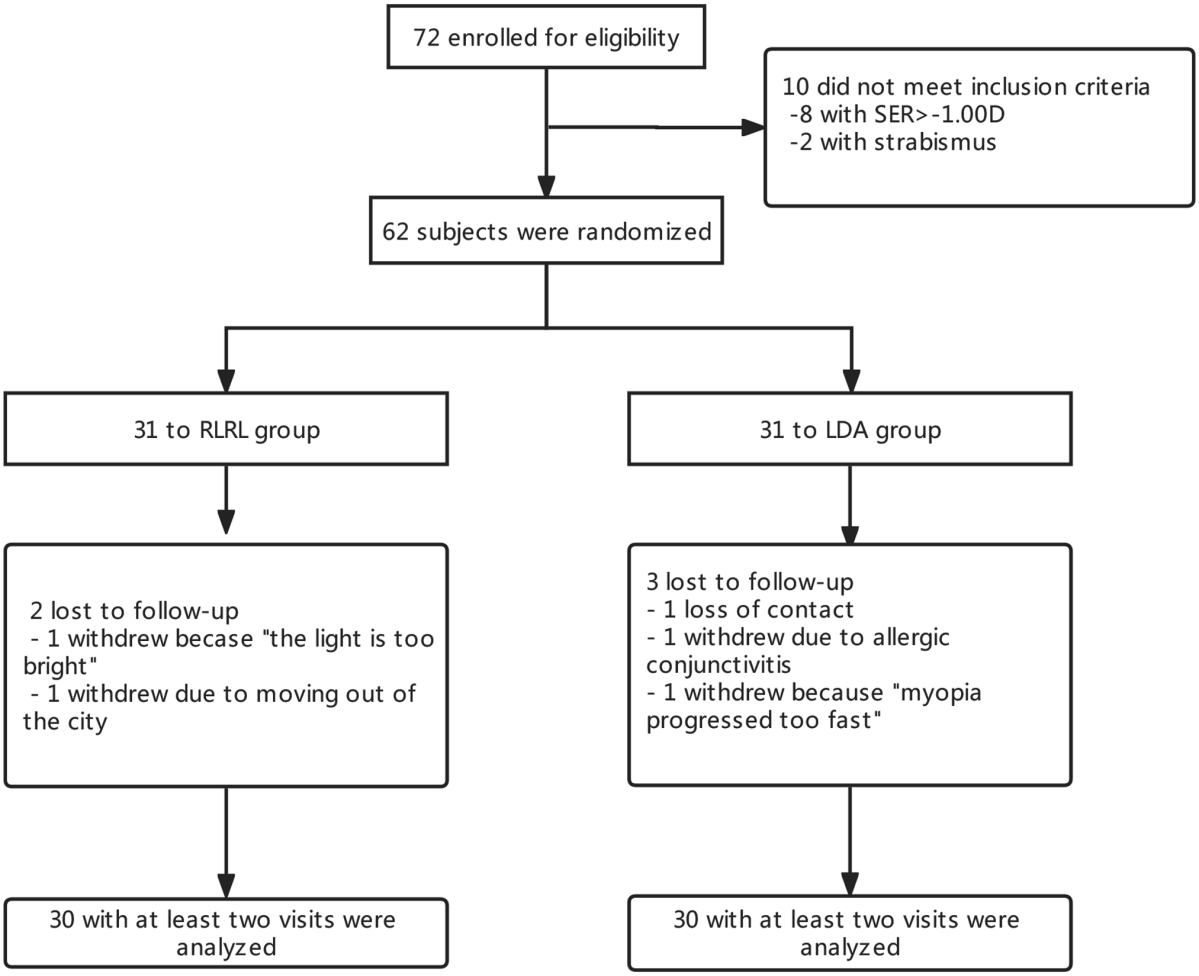
Flow diagram of the study design.

Of the 72 subjects examined for eligibility, 62 children were enrolled in this study. Following randomization, each treatment group had 31 participants (Fig. 1). All participants completed their baseline visit, 57 (91.9%) completed the 12-month visit, and 60 (96.8%) participants completed at least one follow-up visit. Among all participants, the mean age at baseline was 10.04 ± 1.75 years, and 50.0% were female. The baseline demographic and ocular characteristics of participants by treatment group are provided in Table 1. Overall, mean age and gender, baseline SER, AL, and proportion of high myopia (SER ≤ −6.0 D) were similar in RLRL and LDA groups.

**Table 1.** Baseline Characteristics of the Intervention and Control Groups

Baseline Characteristics	RLRL Group (*n* = 31; 62 Eyes)	LDA Group (*n* = 31; 62 Eyes)
Age (y)		
6–9, *n* (%)	18 (58.1)	15 (48.4)
10–15, *n* (%)	13 (41.9)	17 (51.6)
Mean (SD)	9.78 (1.58)	10.31 (1.90)
Female, *n* (%)	17 (54.8)	14 (45.2)
SER (D)		
> −3.0, *n* (%)	43 (69.4)	39 (62.9)
> −6.0, ≤ −3.0, *n* (%)	19 (30.7)	21 (33.9)
≤ −6.0, *n* (%)	0 (0.0)	2 (3.2)
Mean (SD)	−2.60 (1.17)	−2.59 (1.24)
Range	−5.25 to −1.00	−7.00 to −1.00
AL (mm), mean (SD)	24.48 (0.79)	24.67 (0.98)

### Primary Outcome

**Figure 2.**
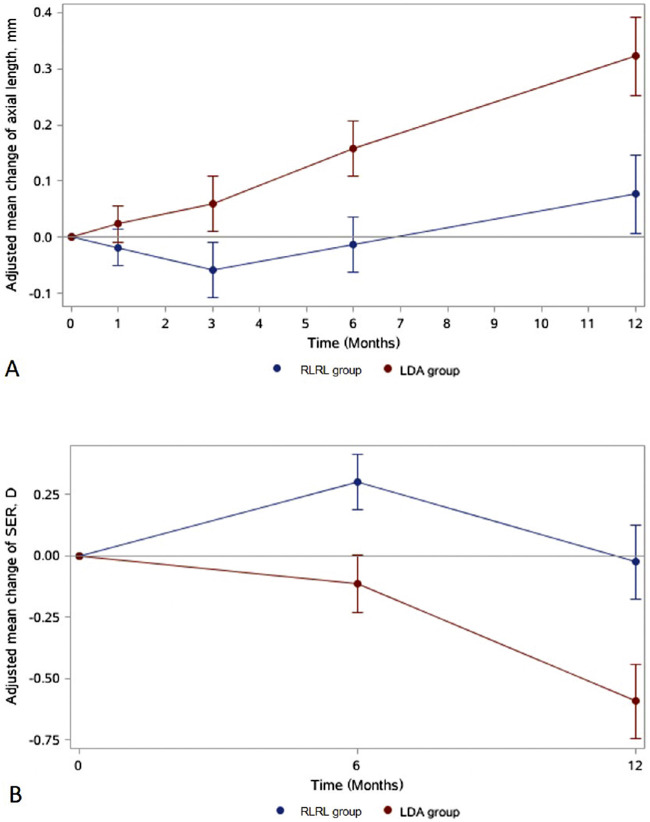
Adjusted changes in AL (A) and SER (B) at each visit for the RLRL and LDA groups.

After adjusting for baseline age, AL, sex, visit, group, and group-by-visit, the 12-month changes in AL were 0.08 mm (95% confidence interval [CI], 0.03–0.14) in the RLRL group and 0.33 mm (95% CI, 0.27–0.38) in the LDA group, with a mean difference (MD) of −0.24 mm (95% CI, −0.32 to −0.17 mm; *P* < 0.001) (Fig. 2, Supplementary Table S1). Axial elongation in the LDA group was larger than that in the RLRL group at the 3-, 6-, and 12-month follow-up (*P* < 0.05) (Fig. 2, Supplementary Table S1). The change over the previous 6 months was also significantly different between the two groups (*P* < 0.001).The longitudinal mixed model indicated that baseline age, group, visits, and group-by-visit interactions (all *P* < 0.05) were significantly associated with change in AL, but sex and baseline AL were not significant risk factors for AL progression (Supplementary Table S2).

### Secondary Outcomes

The change in SER after 12 months was −0.03 D (95% CI, −0.01 to 0.08) in the RLRL group and −0.60 D (95% CI, −0.71 to −0.48) for the LDA group (Fig. 2, Supplementary Table S1). There were significant differences between the RLRL and LDA groups at 6 months (MD = 0.41 D; 95% CI, 0.29–0.54; *P* < 0.001) and 12 months (MD = 0.57 D; 95% CI, 0.40–0.73; *P* < 0.001) (Fig. 2, Supplementary Table S1). The SER changes over the previous 6 months were significantly different between the two groups (*P* = 0.0039). In the longitudinal linear mixed-effects model for SER, the intervention group, visits, and group-by-visit interactions were significant factors for myopia progression (all *P* < 0.05) (Supplementary Table S2).

Changes in ACD, CC, and WTW corneal diameter did not differ significantly over 12 months (all *P* > 0.05) (Supplementary Table S3). Changes in ocular biometry at 1, 3, and 6 months are presented in Supplementary Table S3. At 12 months, the proportion of subjects with a worsening in UCVA of at least two lines was smaller in the RLRL group (3.5% vs. 37.5%), and more children showed two lines of improvement in the RLRL group (67.2% vs. 28.6%) (Table 2). The proportion of children who had a change in UCVA within one line and who maintained a BCVA of 20/20 was similar between the two groups. No ocular hypertension was found during follow-up, and the rate of IOP change ≥5 mmHg from baseline was similar in the two treatment groups (Table 2).

**Table 2.** One-Year Changes in UCVA, BCVA, and Fluctuation in IOP in the RLRL and LDA Groups

One-Year Changes	RLRL Group (*n* = 58 Eyes), *n* (%)	LDA Group (*n* = 56 Eyes), *n* (%)
Change in UCVA		
≥2 lines worsening	2 (3.5)	21 (37.5)
±1 line	17 (29.3)	19 (33.9)
≥2 lines improvement	39 (67.2)	16 (28.6)
Maintained BCVA of 20/20	58 (100.0)	56 (100.0)
Fluctuation of IOP		
≤5 mmHg	53 (91.4)	54 (96.4)
>5 mmHg	5 (8.6)	2 (3.8)

### Degree of Axial Elongation and Myopia Progression

**Figure 3.**
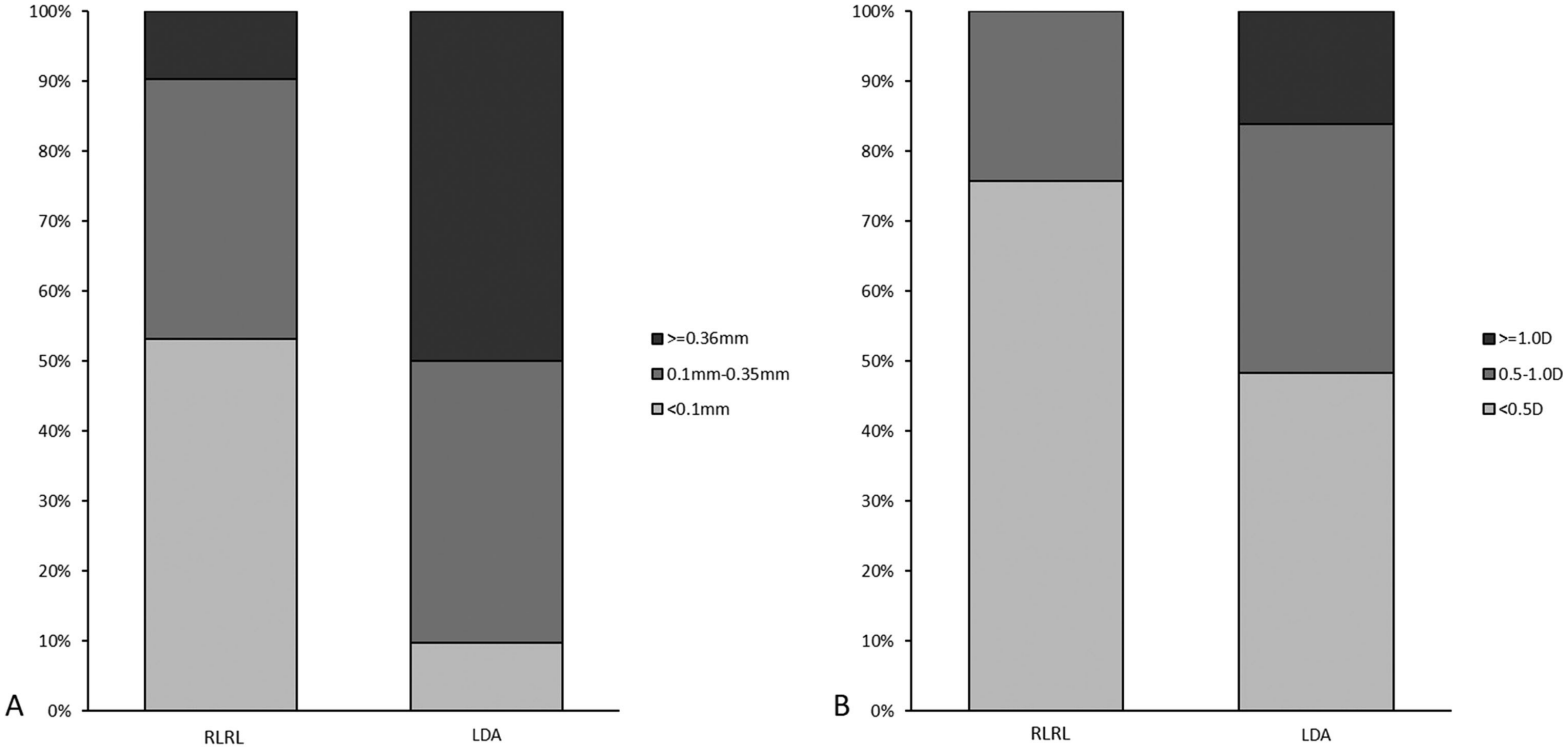
Distribution of changes in AL (A) and SER (B) at 1 year for the RLRL and LDA groups.

Over 12 months, the proportion of subjects who progressed <0.1 mm in AL was higher in the RLRL group (53.2% vs. 9.7%; *P* < 0.001), and fewer subjects progressed ≥0.36 mm in the RLRL group (9.7% vs. 50.0%; *P* < 0.001). Similarly, the percentage of subjects in the RLRL group with progression in SER < 0.5 D was higher compared to the LDA group (75.8% vs. 48.4%; *P* < 0.001), and the percentage was lower for progression of SER > 1 D compared to the LDA group (0.0% vs. 16.1%; *P* < 0.001) (Fig. 3).

We also compared AL shortening (e.g., decrease of AL > 0.05 mm from baseline) between the two treatment groups. RLRL therapy achieved a higher percentage of eyes with AL shortening than did the LDA group at 1 month (42.6% vs. 7.4%; *P* < 0.001), 3 months (50.0% vs. 10.0%; *P* < 0.001), 6 months (31.7% vs. 5.2%; *P* < 0.001), and 12 months (20.6% vs. 3.6%; *P* < 0.001). The mean AL shortening in the first months was −0.08 mm (95% CI, −0.17 to −0.05) in the RLRL group, and it was −0.11 mm (95% CI, −0.14 to −0.08) in the LDA group. At 12 months, the mean AL shortening from baseline was −0.22 mm (95% CI, −0.45 to −0.05) in the RLRL group, and it was −0.19 mm (95% CI, −0.24 to −0.14) in the LDA group.

### Compliance and Treatment Effect

The mean compliance rate was 75.8% (range, 31.9%–97.3%) in the RLRL group and 88.5% (range, 74.5%–6.6%) in the LDA group. The compliance rate was not significantly correlated with 1-year AL change in either the RLRL group (*r* = 0.37, *P* = 0.43) or the LDA group (*r* = −0.05, *P* = 0.71) (Supplementary Fig. S3).

### Adverse Events

No severe adverse event was reported over the 12 months of follow-up. In the RLRL group, one participant dropped out because the brightness of the red light was uncomfortable. Another participant reported dizziness after the red-light therapy, but the symptom resolved after a few minutes and only occurred for a few days. In the LDA group, one participant reported photophobia within the first month of treatment, one participant stopped treatment due to allergic conjunctivitis, and one participant discontinued atropine treatment, complaining that “myopia progressed too fast.” No structural damage in the macular was observed from optical coherence tomography images in any of the subjects.

## Discussion

This RCT compared RLRL therapy with LDA (0.01% atropine) for myopia control and found that RLRL significantly slowed axial elongation over 3, 6, and 12 months compared with LDA. After 12 months of therapy, the MD between RLRL and LDA treatment was −0.24 mm for AL elongation, and it was 0.57 D for myopia progression. Our results provide new evidence that RLRL is a better intervention than LDA for myopia control.

### Valid Treatment Efficacy of Red-Light Therapy

The efficacy of RLRL has been reported in previous studies, with AL progression following 6 months of use ranging from −0.06 mm to 0.04 mm at 6 months and 0.13 mm at 12 months.^16^^–^^18^ This study result aligns with previous findings, with AL changes of −0.01 mm at 6 months and 0.08 mm at 1 year in the RLRL group. Refraction changes were also similar, with SER changes of 0.29 D at 6 months and 0.05 D at 6 to 12 months, similar to the results for the studies by Jiang et al.^16^ (−0.03 D and 0.20 D, respectively) and Zhou et al.^17^ (0.19 D at 6 months). The continuity of such promising results reinforces the notion that RLRL is an effective treatment for AL and refraction in myopes.

### First RCT to Compare Red Light With Atropine

To the best of our knowledge, this is the first RCT to compare the treatment effect of RLRL to 0.01% atropine eye drops. LDA has little influence on pupil size and provides a smaller rebound effect compared with higher doses, which has increased the popularity of atropine in recent years.^21^ Despite this, results from the current study and those of previous studies indicate that LDA has limited control for AL elongation.^11^^,^^22^^,^^23^ Given the concentration-dependent response of atropine, a 1% dose is necessary to effectively control AL, but such a dose increases the risks of adverse events such as allergic reactions, glare, and blurred near vision.^9^ Our results indicate that RLRL is a more effective alternative treatment modality than 0.01% atropine eye drops, as we found that it offered better myopia control and fewer side effects during the first 6 months, as well as during the last 6 months.

In the current study, LDA achieved comparable changes in AL and SER as the previously reported findings. Our 12-month changes in SER and AL were −0.58 D and 0.31 mm, respectively, comparable to the LAMP study using the same dose of atropine (−0.59 D and 0.36 mm, respectively)^11^ and the ATOM2 study (−0.54 D and 0.30 mm, respectively).^22^ In the LDA group in the LAMP study, the percent of subjects with SER progression less than 0.5 D was 43.8%, a finding similar to ours (48.4%), although the percentage of those with myopia progression > 1.0 D was lower in the current study than in the LAMP study (16.1% vs. 27.8%, respectively). Thus, the treatment efficacy of 0.01% atropine treatment in the current study is consistent with previous findings and provides a reliable comparison for determining the efficacy of RLRL therapy.

### AL Shortening in Treatment

AL shortening is a phenomenon that is rarely reported following myopia control treatments. Short-term decreases in AL have been reported following orthokeratology treatment,^24^^,^^25^ and persistent AL shortening was reported in a 4-year-old child given violet light-transmitting eyeglasses, which led to −0.20 AL over 2 years.^26^ In the ATOM1 study, AL shortened −0.14 mm in subjects using 1% atropine eye drops, but the proportion and range were not reported.^9^ In this study, clinically significant AL shortening was observed in fewer than 10% of subjects using 0.01% atropine but this was even more common in the RLRL group (20.6%), consistent with three previous studies reporting that RLRL decreased AL over 6 months in a large proportion of children.^16^^–^^18^ Similar to our findings, Jiang et al.^16^ reported that AL decreased by −0.05 to −0.03 mm in the first month, and the proportion of persistent AL shortening was as much as 21.6% at 12 months. The mechanism for AL shortening is not clear, although choroid thickening has been observed following RLRL.^16^^–^^18^ Despite this, the magnitude of AL shortening cannot be fully explained by choroid thickening. It is instead speculated that red light increases blood flow to the choroid, influencing sclera remodeling additionally.^27^^,^^28^ Red light can also improve human photoreceptor function by changing thresholds for tritan and protan function,^29^ which may modulate the metabolism of the fundus. Further investigations are required to understand the exact mechanism of red-light therapy and its long-term effects on human eyes.

### Compliance and Dose–Response Effect

In contrast to Jiang et al.,^16^ no significant association was found between myopia progression and compliance with RLRL therapy. Furthermore, a dose–response effect of RLRL is not clear in the current study due to insufficient follow-up. It should be noted that we instructed participants to conduct RLRL therapy every day, similar to the studies by Xiong et al.^18^ and Zhou et al.,^17^ but in the Jiang et al.^16^ study the protocol was only 5 days per week. Considering that the study by Jiang et al.^16^ had the largest myopia progression among currently published RLRL studies, this could infer a dose-dependent effect. Evidence is still in its infancy on RLRL, and optimal treatment frequency and dose–response effects using 650-nm red light for myopia control must be investigated further.

### Study Strengths and Limitations

Strengths of this study included its RCT design, high response rate (>85% at each visit), and the comparison between a new intervention and a widely used treatment. Nonetheless, several limitations should be acknowledged. First, reports of compliance with the atropine treatment were less reliable compared with those for the RLRL therapy. The RLRL therapy had a built-in server that monitored compliance but compliance with the LDA therapy was determined by the number of empty bottles. We could not avoid the deliberate discarding of eye drops or false reporting from parents; therefore, the higher compliance reported for LDA should be interpreted with caution. Second, this study was a single-center Chinese study, and ideally a multicenter study that investigates the efficacy of RLRL in other geographic regions should be conducted to confirm these findings. Third, only 0.01% atropine was used as comparison, but in reality different doses of atropine eye drops can be used for myopia control. Fourth, due to the COVID-19 quarantine policy in China, all of the participants began online learning at the eighth month of the study. A comparison of the between-visit change is thus not available in this study. Finally, follow-up was only 12 months, and a longer follow-up is needed to confirm whether RLRL has cumulative benefits with longer use.

In conclusion, this RCT determined that the myopia control efficacy of RLRL is superior to that of LDA, as it found that RLRL was a more effective treatment for controlling AL and myopia progression over 12 months. Both treatments are well tolerated without severe adverse events; thus, RLRL therapy should be considered as a promising treatment for myopia control in children.

## Supplementary Material

Supplement 1

Supplement 2
