# Supplementary material for: Efficacy Comparison of Repeated Low-Level Red Light and Low-Dose Atropine for Myopia Control: A Randomized Controlled Trial
Source: Transl Vis Sci Technol. 2022 Oct 21;11(10):33. doi: 10.1167/tvst.11.10.33 (PMC9617501; doi:10.1167/tvst.11.10.33)
Supplement: Supplement 1 [file tvst-11-10-33_s001.pdf]

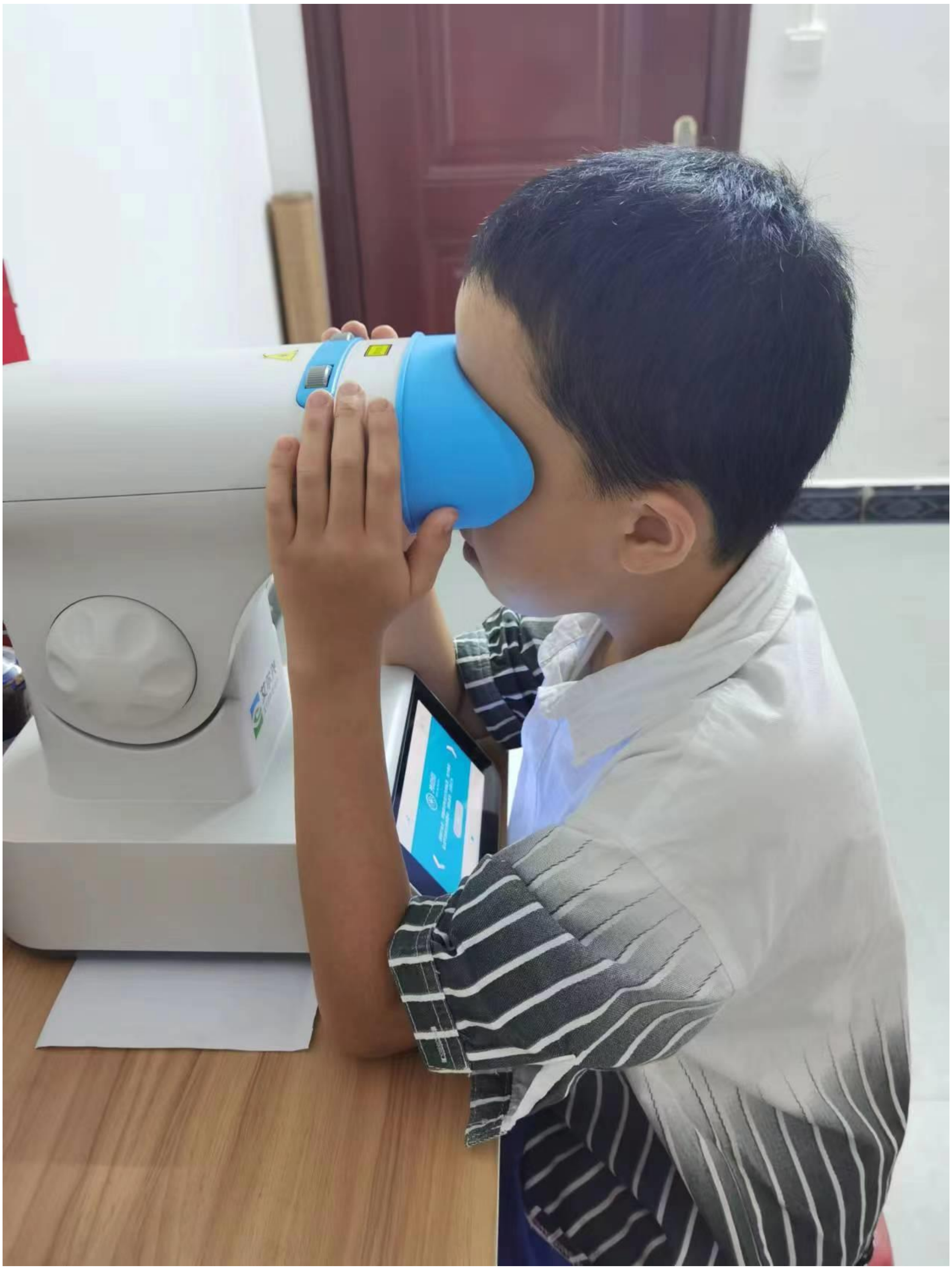

Figure S1. Repeated Low-level Red Light therapy.

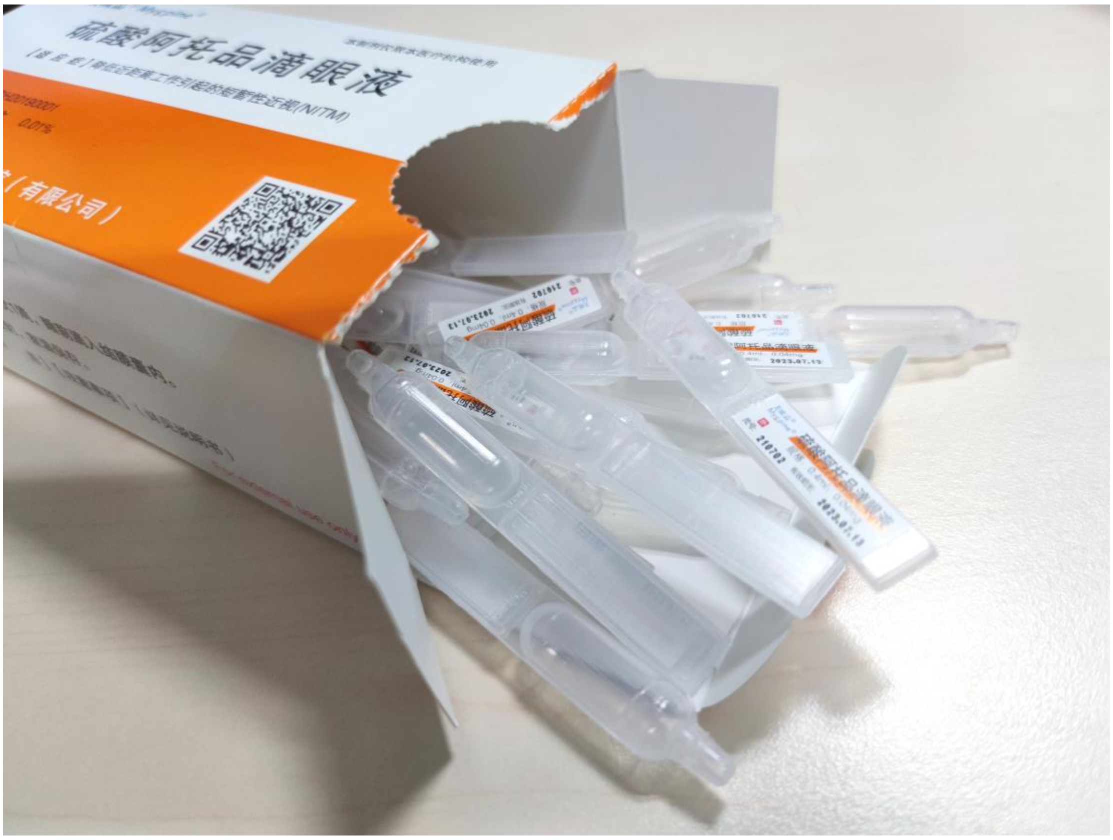

Figure S2. Used containers brought by subjects at follow-up visit.

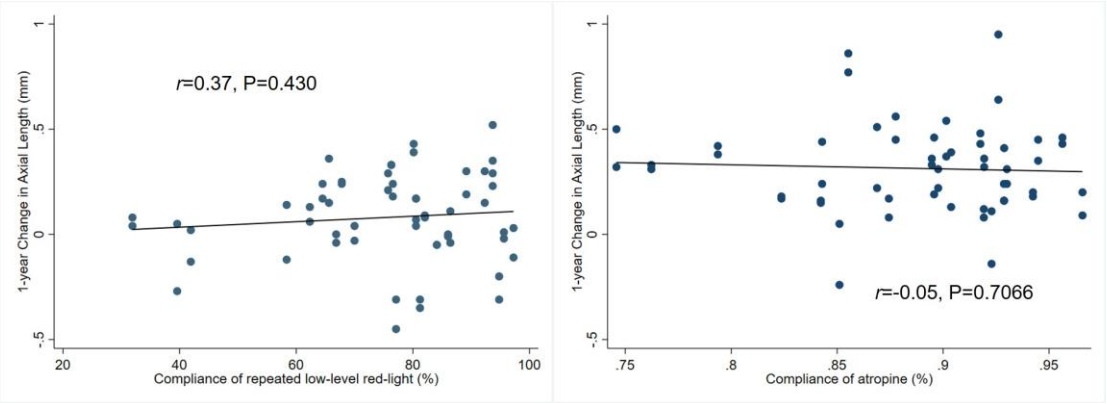

Figure S3. Left: compliance of repeated low-level red light therapy with 1-year change in axial length. Right: compliance of low-dose atropine eye drops with 1-year change in axial length.
