# Supplementary material for: Efficacy Comparison of Repeated Low-Level Red Light and Low-Dose Atropine for Myopia Control: A Randomized Controlled Trial
Source: Transl Vis Sci Technol. 2022 Oct 21;11(10):33. doi: 10.1167/tvst.11.10.33 (PMC9617501; doi:10.1167/tvst.11.10.33)
Supplement: Supplement 2 [file tvst-11-10-33_s002.pdf]

Table S1. Adjusted changes in axial length and refraction from baseline to 12 months in the low-level red light group and low-dose atropine group.

Variable		RLRL group ,Mean(95%CI)		LDA group, Mean(95%CI)	Difference, Mean(95%CI)
**Primary outcome**					
**Change in AL, mm**	N		N		
**1 month**	54	-0.02(-0.05, 0.01)	54	0.02(-0.01, 0.05)	-0.04(-0.09, 0.00)
**3 months**	60	-0.06(-0.10, -0.02)	60	0.06(0.01, 0.10)	-0.12(-0.18, -0.06)*
**6 months**	60	-0.01(-0.05, 0.02)	58	0.16(0.12, 0.19)	-0.17(-0.22, -0.12)*
**12 months**	58	0.08(0.03, 0.14)	56	0.33(0.27, 0.38)	-0.24(-0.32, -0.17)**
**Secondary outcome**					
**Change in SER, D**	N		N		
**6 months**	60	0.04(0.22, 0.39)	58	-0.11(-0.19, -0.02)	0.41(0.29, 0.54)**
**12 months**	58	-0.03(-0.01, 0.08)	56	-0.60(-0.71, -0.48)	0.57(0.40, 0.73)**

RLRL: repeated low-level red light. LDA: low-dose atropine. CI: confidence interval. AL: axial length. SER: spherical equivalent.

*P<0.05

**P<0.001

Table S2. Mixed model adjusting factors of change in axial length and cycloplegic spherical equivalent

Factor	Coefficient (SE)	P
**Change in AL as outcome**		
Age, year	-0.01(0.01)	0.046
Male	0.02(0.02)	0.44
Baseline AL, mm	-0.01(0.01)	0.42
Treatment Group (RLRL vs. LDA)	-0.24(0.04)	<0.001
1-month visit	-0.03(0.03)	<0.001
3-month visit	-0.03(0.02)	<0.001
6-month visit	-0.17(0.01)	<0.001
RLRL group* 1-month visit	0.20(0.04)	<0.001
RLRL group* 3-month visit	0.13(0.03)	0.0002
RLRL group* 6-month visit	0.07(0.02)	<0.001
**Change in SER as outcome**		
Age, year	0.03(0.02)	0.15
Male	0.04(0.06)	0.50
Baseline SER, D	0.03(0.03)	0.71
Treatment Group (RLRL vs. LDA)	0.57(0.08)	<0.001
6-month visit	0.49(0.04)	<0.001
RLRL group* 6-month visit	-0.15(0.05)	0.004

SE: standard error. SER: spherical equivalent. AL: axial length. RLRL: repeated low-level red light. LDA: low-dose atropine.

Table S3. Adjusted changes in corneal curvature, anterior chamber depth and white-to-white diameter from baseline to 12 months in the low-level red light group and low-dose atropine group

Variable		RLRL group ,Mean(95%CI)		LDA group, Mean(95%CI)	Difference, Mean(95%CI)
**Change in CC, D**	N		N		
**1 month**	54	0.05(0.01, 0.09)	54	0.01(-0.03, 0.0.06)	0.04(-0.02, 0.10)
**3 months**	60	0.03(-0.07, 0.13)	60	0.01(-0.09, 0.11)	0.02(-0.12, 0.16)
**6 months**	60	-0.04(-0.09,0.00)	58	-0.03(-0.08, 0.01)	-0.01(-0.08, 0.05)
**12 months**	58	0.01(-0.03, 0.05)	56	-0.01(-0.05, 0.04)	0.02(-0.04, 0.07)
**Change in ACD, mm**					
**1 month**	54	0.01(-0.02, 0.03)	54	0.05(0.03, 0.08)	-0.05(-0.09, -0.01)*
**3 months**	60	0.00(-0.05, 0.04)	60	0.06(0.03, 0.09)	-0.01(-0.07, 0.05)
**6 months**	60	0.01(-0.02, 0.04)	58	0.06(0.03, 0.09)	-0.05(-0.09, -0.01)*
**12 months**	58	0.03(0.00, 0.06)	56	0.07(0.04, 0.10)	-0.04(-0.09, 0.00)
**Change in WTW, mm**					
**1 month**	54	-0.01(-0.08, 0.07)	54	0.01(-0.06, 0.09)	-0.02(-0.13, 0.08)
**3 months**	60	-0.03(-0.10, 0.04)	60	0.02(-0.05, 0.09)	-0.05(-0.15, 0.05)
**6 months**	60	-0.02(-0.10, 0.04)	58	0.03(-0.05, 0.10)	-0.05(-0.15, 0.06)
**12 months**	58	0.03(-0.04, 0.10)	56	0.06(-0.01, 0.14)	-0.03(-0.14, 0.07)

RLRL: repeated low-level red light. LDA: low-dose atropine. CI: confidence interval. CC: corneal curvature. ACD: anterior chamber depth. WTW: white-to-white.

*P<0.05
